# Early Recognition of High Risk of Bipolar Disorder and Psychosis: An Overview of the ZInEP “Early Recognition” Study

**DOI:** 10.3389/fpubh.2014.00166

**Published:** 2014-10-01

**Authors:** Anastasia Theodoridou, Karsten Heekeren, Diane Dvorsky, Sibylle Metzler, Maurizia Franscini, Helene Haker, Wolfram Kawohl, Nicolas Rüsch, Susanne Walitza, Wulf Rössler

**Affiliations:** ^1^Department of Psychiatry, Psychotherapy and Psychosomatics, University Hospital of Psychiatry Zurich, Zurich, Switzerland; ^2^The Zurich Program for Sustainable Development of Mental Health Services (ZInEP), University Hospital of Psychiatry Zurich, Zurich, Switzerland; ^3^University Hospital of Child and Adolescent Psychiatry, University of Zurich, Zurich, Switzerland; ^4^Translational Neuromodeling Unit, University of Zurich and ETH Zurich, Zurich, Switzerland; ^5^Collegium Helveticum, Zurich, Switzerland

**Keywords:** bipolar disorder, psychosis, early recognition, high risk, ZInEP

## Abstract

Early detection of persons with first signs of emerging psychosis is regarded as a promising strategy to reduce the burden of the disease. In recent years, there has been increasing interest in early detection of psychosis and bipolar disorders, with a clear need for sufficient sample sizes in prospective research. The underlying brain network disturbances in individuals at risk or with a prodrome are complex and yet not well known. This paper provides the rationale and design of a prospective longitudinal study focused on at-risk states of psychosis and bipolar disorder. The study is carried out within the context of the Zurich Program for Sustainable Development of Mental Health services (Zürcher Impulsprogramm zur Nachhaltigen Entwicklung der Psychiatrie). Persons at risk for psychosis or bipolar disorder between 13 and 35 years of age are examined by using a multi-level-approach (psychopathology, neuropsychology, genetics, electrophysiology, sociophysiology, magnetic resonance imaging, near-infrared spectroscopy). The included adolescents and young adults have four follow-ups at 6, 12, 24, and 36 months. This approach provides data for a better understanding of the relevant mechanisms involved in the onset of psychosis and bipolar disorder, which can serve as targets for future interventions. But for daily clinical practice a practicable “early recognition” approach is required. The results of this study will be useful to identify the strongest predictors and to delineate a prediction model.

## Introduction

Early detection and prevention is considered an essential strategy to reduce years lived with disability (YLD) and reduce the stigma attached to mental disorders (1). However, in some people the initial symptoms are not properly recognized (2, 3). Lack or delay of treatment can lead to an unfavorable or chronic course of the illness (4, 5). Research on the psychosis risk syndrome, which aims to reduce transition rates to psychosis, presents a challenge to current ascertainment and intervention methods (6). Some at-risk individuals do not convert to psychosis, but have outcomes that fall within the spectrum of psychotic disorders. Therefore, it is necessary to improve knowledge about prediction. Currently, new methodological approaches such as multivariate modeling and machine learning methods are discussed in regard to the prospects and caveats (7–10). At the same time, the field is moving toward extending efforts to study the bipolar disorder risk syndrome (11).

The first phase of research into early recognition of psychosis tended to favor specific assessment tools, definition of high-risk (HR) criteria and outcomes as well as the application of these methods (11–14). Even if the duration of untreated psychosis (DUP) has been shortened in the last years, an average of 1 year DUP in highly developed health care systems remains (15). But once basic symptoms and “ultra-high risk” (UHR)-symptoms are detectable, associated pathophysiology may have already progressed (16). Assuming that the brain is a complex structure in which anatomical and functional connectivity occurs at many levels (17), that plasticity occurs during all stages of development and that environmental factors can also have important effects on brain functions, psychiatry must take various levels of evidence into account (18). To detect this underlying pathophysiology, putative biomarkers, and intermediary phenotypes need to be developed and tested in a sufficiently large cohort with members truly at risk for psychosis (14). The diagnostic process of bipolar disorder is made more difficult, especially in young people, by complex diagnostic conditions from which bipolar disorder must be distinguished and also from the occurrence of adverse life circumstances, all of which do not facilitate the diagnostic process (19). A combination of clinical risk factors with precursors and family-risk could improve early identification of bipolar disorder (20). Even though there is evidence suggesting that the characterization of early phases in the development of the bipolar disorder is viable further evaluation in larger, prospective studies is needed (21–24).

There is evidence for a substantial overlap in genetic susceptibility to bipolar disorder and schizophrenia but also for the existence of non-shared genetic risk factors (25–27). However, attenuated positive symptoms occur in both the schizophrenic and the bipolar prodromal phase (22, 28, 29). Potential analogy between the genetic and phenotypic overlap between both phases raises the question about the nature of the at-risk phase of different mental disorders.

Previous research into the prodromal phase of first-episode psychosis has brought knowledge regarding the development of psychosis (30). But until now it is unclear to what extent the findings from research into the prodromal phase of first-episode psychosis reflect psychiatric distress in general and to what extent they are specifically associated with the development of a full-blown psychotic disorder (30). It may be that a more general strategy for early intervention in a range of mental disorders could be a useful approach. Many disorders could develop from initial non-specific symptoms and syndromes, i.e., from a background of specific and non-specific risk factors (31). Understanding the interplay of developmental factors and pathophysiological changes will help to study the moderating risk and resilience factors.

Main objective of the ZInEP “early recognition” study is to develop a risk model to estimate conversion risk and to specify how environmental and pathophysiological factors affect functioning. We hypothesize that our defined at-risk groups for psychosis and bipolar disorder could be distinguished based on the neurocognitive and neurobiological measures. In this paper, we present an overview of the study methodology and the characteristics of the sample recruited so far.

## Materials and Methods

### Study design

The Zurich Early Recognition Program [part of The Zurich Program for Sustainable Development of Mental Health services (Zürcher Impulsprogramm zur nachhaltigen Entwicklung der Psychiatrie; www.zinep.ch)] is based in a catchment area in the Canton Zurich, Switzerland with approximately 1,300,000 inhabitants. Our study has a longitudinal multi-level design with a baseline assessment and four follow-up examinations at 6, 12, 24, and 36 months or if transition to full-blown psychotic or affective disorder occurs.

### Referral and recruitment

All persons in the indicated age range (subjects between 13 and 35 years of age) presenting to any of our four study centers, situated in urban area, and embedded into established early recognition units, were screened for the presence of the inclusion criteria. Psychiatrists, child and adolescent psychiatrists, psychologists, general practitioners, outreach clinics, counseling services, teachers, and affected persons or their worried family members could refer to the early recognition units (hospital based units employing standardized criteria to identify persons at risk for psychosis or bipolar disorder and offering appropriate counseling). We provided information about the study through local workshops, articles in professional journals, flyers, and a study-website. If at least one inclusion criterion was fulfilled and the participants gave written informed consent, the baseline examination including clinical (including blood analysis), psychopathological, neurocognitive, neurophysiological, genetic, sociophysiological, and magnetic resonance (MR) measures were performed. We also recruited normal control subjects from the community by way of advertisements. They were healthy volunteers, aged 13–35 years, without any personal history of psychiatric illness including schizophrenia, bipolar, or other psychotic disorders.

### Participants

For inclusion, participants had to fulfill at least one of the following three criteria (listed in Table 1): (i) HR status for psychosis assessed by the adult (14) or children-youth (32, 33) version of the Schizophrenia Proneness Interview, with at least one cognitive–perceptive basic symptom or at least two cognitive disturbances; or (ii) ultra-HR status (UHR) for psychosis as rated by the Structured Interview for Prodromal Syndromes (SIPS) (12, 34) with at least one attenuated psychotic symptom, or at least one brief limited intermittent psychotic symptom, or state-trait criteria [reduction in global assessment of functioning (GAF) of >30% in the past year plus either schizotypal personality disorder or a first degree relative with psychosis]; or (iii) risk of bipolar disorder (at-risk bp), defined by a score ≥14 in the hypomania checklist, a self-report measure of life-time hypomanic symptoms (35).

**Table 1:** **Risk groups**.

**High-risk (HR) for psychosis**
at least one cognitive–perceptive basic symptom or
at least two cognitive disturbances
**Ultra-high-risk (UHR) for psychosis**
at least one attenuated psychotic symptom or
at least one brief limited intermittent psychotic symptom or
state-trait criteria (reduction in global assessment of functioning
of >30% in the past year plus either schizotypal personality disorder
or a first degree relative with psychosis)
**Risk of bipolar disorder (at-risk bp)**
defined by a score ≥14 in the Hypomania Checklist

Exclusion criteria for study participation were diagnosed schizophrenic, substance-induced or organic psychosis, other symptomatic organic mental disorders, manifest bipolar disorder, current substance, or alcohol dependence, age below 13 or above 35 years, incapacity to consent, e.g., due to an acute and severe psychopathological state or low intellectual abilities with IQ <80. Axis-I comorbidity was assessed by the Mini-International Neuropsychiatric Interview based on DSM-IV criteria (36). The presence of psychotic symptoms for more than 1 week was assessed. Transition to psychosis or bipolar disorder was defined according to ICD-10 criteria.

### Informed consent

Adult and young persons were informed about the study by a research-psychologist or research-psychiatrist by means of a comprehensive information letter. When all the information on the trial was understood, the consent form was signed (up to age 18 by both the adolescent and their parents) and the person was invited for the first screening. Participants could always withdraw from participating in the study. The study was approved by the local ethics committee and conducted in accordance with the guidelines of the Helsinki Declaration.

### Assessments

A multi-level assessment of psychopathology, neurocognitive, neurophysiological, genetic, structural, and functional brain abnormalities is carried out.

Information on psychopathology was gathered with the Schizophrenia Prediction Instrument-adult (14) and -child and youth Version (SPI-CY, SPI-A) (32, 33), the SIPS (12, 34), the Positive and Negative Symptom Scale (PANSS) (37), Hypomania Checklist (HCL-32) (35), Calgary Depression Scale (38), Hamilton Depression Scale (HAMD) (39), and Beck Anxiety Inventory (BAI) (40).

Data about the socio-demographic background, physical health, obstetric and family history, premorbid adjustment, functioning and disability by psychiatric symptoms, daily hassles stress, and quality of life were collected via: Obstetric Complications Scale (OCS) (41), Clinical Global Impression Rating Scales (CGI) (42), GAF (43), Hopkins Symptom Checklist (SCL-90-R) (44), Camberwell Assessment of Need (CAN) (45), the General Self-Efficacy-Scale (GSE) (46), Daily Hassles and Stress Scale (DHSS) (47), Manchester Short Assessment of Quality of Life (MANSA) (48). All investigators were either psychologists or psychiatrists who received extensive training.

### Neurocognitive assessments

A set of well-established neuropsychological tests was administered in accordance with the MATRIC Consensus Cognitive Battery (49–51). Verbal IQ was estimated with a word recognition test (MWT-B) for adults (52) and a test of receptive vocabulary for adolescents (PPVT) (53). Abstract reasoning abilities were estimated using a non-verbal task (scale 3 of LPS) (54). Measures of attention were assessed by Continuous Performance Test (CPT-OX) (55), a test of selective attention (FAIR) (56), the Stroop Test (57), and the divided attention subtest of a computer-administered test series (TAP) (58). The Trail Making Test Parts A and B (59) were used to assess psychomotor speed, attention, and cognitive flexibility.

Measures of verbal and figural learning and memory were collected from a German Auditory Verbal Learning Test (60, 61) and from the Rey Visual Design Learning Test (57).

Measures of executive function were provided by a test of verbal (RWT) and figural (five-point test) fluency (62, 63), working memory by subtests of the Wechsler Adult Intelligence Scale (WIE) (64), a computer-administered Wisconsin Card Sorting Test (CKV) (65), and Tower of Hanoi (ToH) (66).

### Molecular genetic studies

Genetic and epigenetic studies will be carried out in the Neurobiochemistry Laboratory of the University Hospital of Child and Adolescent Psychiatry (E. Grünblatt, S. Walitza) together with the Institute of Medical Genetics, University of Zurich (A. Rauch). Peripheral blood samples were collected from at-risk individuals for DNA and RNA (PAXgene Blood RNA Tubes) analysis. For gene expression profiling, RNA was reverse-transcribed into cDNA using the iScript cDNA synthesis kit. Using quantitative real-time RT-PCR, the profile of transcript for various genes was investigated. DNA is available from 218 participants, of whom 2/3 are adolescents while the other 1/3 are young adults. Various candidate gene variations (SNPs and polymorphism) and gene expression analyses have been performed up to now (e.g., d-amino acid oxidase activator, dopamine transporter, neuroregulin). Genome wide CNV analysis in at-risk persons for psychosis and bipolar disorders is still on-going.

### Magnetic resonance imaging

Images were collected on a Philips Achieva 3 Tesla magnetic resonance imaging (MRI) scanner equipped with an 8-channel standard head coil. The MR-images of all participants were rated by the Department of Neuroradiology at the University of Zurich for exclusion of a visible organic brain disease.

The intended analyses of the MRI data include voxel based morphometry (VBM) and diffusion tensor imaging (DTI) to detect alterations of the cortical structure and the white matter in individuals at-risk. Intrinsic functional connectivity is examined based on resting state functional MRI data (6-min runs). In addition, a reward task [Monetary Incentive Delay Task (67)] was performed to identify possible dysregulations of the dopaminergic system by investigating the neural responses to reward expectation and reward outcomes.

### Clinical neurophysiology

EEG data are recorded with a BrainAmp 32 channel-amplifier. Brain Vision Recorder is used as the recording software (both Brain Products GmbH, Munich, Germany). Silver/silver-chloride-electrodes attached to nylon caps [BrainCap with 32 channels (Easycap, Herrsching-Breitbrunn, Germany)] in accordance with the international 10%-system are applied to the scalp. Impedances are strictly kept below 10 kΩ. EEG Channels are referenced to FCz.

The following electroencephalographic paradigms were used in the study.

#### Somatosensory evoked potentials

Human median nerve somatosensory evoked potentials (SEP) provide the possibility of investigating thalamocortical and early cortical processing (68). SEP of the median nerve show brief high-frequency oscillations (~600 Hz), which underlie the primary cortical low-frequency negative peak 20 ms after stimulation. Altered latency and amplitude of SEP have been shown to be present in schizophrenia (69).

#### Loudness dependence of auditory evoked potentials

The loudness dependence of auditory evoked potentials (LDAEP) is considered to be an indicator of the brain’s serotonergic functioning. Moreover, there are hints of influences of other neurotransmitters than serotonin (70, 71). The LDAEP is altered in patients with schizophrenia (72–75). Stimulus tones of five intensities (60, 70, 80, 90, and 100 dB) were presented.

#### Mismatch negativity

The mismatch negativity (MMN) is supposed to reflect an automatic, preattentive process for change detection (76, 77). A dysfunction in the glutamatergic receptor system is considered to play an important role in schizophrenia-related deficits in MMN (78, 79). In the present study, the MMN paradigm includes changes in frequency, intensity, and duration of tones in repetitive acoustic stimulation.

#### NoGo anteriorization

For the investigation of response control, i.e., the execution (Go) and the inhibition (NoGo) of an anticipated motor response, the continuous performance test (CPT) (55) was used. The gravity center (centroid) of the ERP elicited during the NoGo-condition is located more anteriorly as compared to the Go-condition in healthy subjects and this effect has been reported to be smaller in patients with schizophrenia (80, 81).

#### Near-infrared spectroscopy

Changes in frontal oxygenation parameters O2Hb and HHb are measured with a 52-channel near-infrared spectroscopy-system, Optical Topography^®^ System (Hitachi ETG-4000), light sources within this system are 18 semiconductor lasers of 695 and 830 nm wavelength. For the positioning of the fibreoptics probes with distances of 30 mm between signal and detector are used (both Hitach Medical Corporation, Tokyo, Japan). Data sampling rate is set as 10 samplings per second. Measurement channels cover large areas of the prefrontal cortex, motor, and premotor regions and superior temporal cortex.

Cognitive paradigms employed in the neurophysiological examinations included the Verbal-Fluency Test (semantic and phonemic) performed according to Ehlis et al. (82) and Hermann et al. (83), and the emotional Stroop Test (84, 85).

### Sociophysiology and social cognition

Motor empathy was assessed using the Resonance Test (86, 87). In this behavioral paradigm, contagion by yawning and laughing is rated while participants view short video sequences of yawning and laughing faces.

Different aspects of visual emotion recognition in faces were assessed using several items of the “University of Pennsylvania Computerized Neuropsychological Testing Systems” (88): Penn Facial Memory Test (89), Penn Emotion Recognition Task (90), Penn Emotion Discrimination Task (91), and Penn Facial Emotion Acuity Task (92).

Visual recognition of complex emotions and intentions was assessed using the reading the mind in the eyes task (93).

Social attribution style according to the theory of Kelley and Levine (94) was assessed including a paradigm developed by Rössler and Lackus (95).

The ability to differentiate between self and other on the level of visual feedback to motor actions was assessed using a source monitoring or agency task (96, 97).

Subjective experience of empathic abilities was assessed using the Interpersonal Reactivity Index 28-item Self-Report Questionnaire that comprises four subscales: fantasy scale, empathic concern, perspective taking, and personal distress (98, 99).

### Stigma measures

Perceived stigma was assessed using the 12-item Perceived Devaluation-Discrimination Questionnaire (100). Three items measured perceived legitimacy of discrimination or whether participants felt discrimination against people with mental illness was fair (101, 102). General self-esteem was examined using the 10-item Rosenberg Self-Esteem Scale (103). The desire for social distance from people with mental illness was measured using a 5-item Social Distance Scale (104). We used eight items to assess the cognitive appraisal of stigma as a stressor (105, 106), adapted from Kaiser and colleagues (107). The perception of people with mental illness as a distinct and coherent group in society (entitativity) was measured using four items (108). Group values were assessed by two items on whether respondents felt people with mental illness were a good group in society. The level of identification with the group of people with mental illness was examined by five items (101, 109).

### Sample

In 28 months, 305 persons were screened. Eligible participants (*n* = 273) gave informed consent to participate in the study; 52 withdrew their consent before baseline examination was completed. Therefore, 221 persons entered the study group, of whom 133 (60.2%) were male. The mean age of the sample was 20.99 (±6.0) years (range 13–35 years, median 20 years) with no significant difference between males (21.25 ± 6.1 years) and females (20.60 ± 5.7 years).

Among the 221 subjects, most reported COPER (70.1%), COGDIS (53.4%), and APS (44.3%). Genetic risk plus reduced functioning criterion (7.2%) as well as BLIPS (3.6%) were reported noticeably less frequently. Further, 155 (70.1%) subjects showed 14 or more symptoms on the Hypomania Checklist (HCL-32) and 133 (60.2%) had a score of 12 or more on the HAMD. Also, 147 (66.5%) subjects had a score of 10 or more on the BAI.

Of the subjects, 28.1% met only one inclusion criterion, 43.8% met two and 28.1% met all three inclusion criteria (HR, UHR, at-risk bp). Only a small group of 3.2% (7 subjects) was included on the basis of cognitive disturbances alone.

Among the 221 participants, 81 (36.7%) fulfilled high risk and 107 (48.4%) UHR criteria for psychosis, 155 (70%) fulfilled risk criteria for bipolar disorder.

## Discussion

During the last decades, there has been a considerable increase of Early Recognition and Intervention services. While “HR” studies in psychosis are numerous “HR” research in bipolar disorder are emerging. Conceptualization of early recognition of at-risk phases of psychosis and bipolar disorder is complicated due to the mix of heterogeneity and overlap regarding genetic and phenomenological aspects. The ZInEP “early recognition” longitudinal study aims to better understand the predictors of psychosis/bipolar disorder onset and mechanisms for the development of both disorders.

Focusing on both the psychosis at-risk and the bipolar at-risk group, we confirm an overlap between the different at-risk groups.

Recently, the European Prediction of Psychosis Study (EPOS) group suggested a 2-step risk assessment, with UHR and cognitive disturbance criteria for general risk and the prognostic scores as a second-step tool for further risk classification. They propose testing a multi-level model including additional neurocognitive, neurobiological, socio-biographical, and environmental variables to see if this model increases predictive accuracy (8). Similarly to EPOS and contrary to NAPLES (110) our group shows a lower proportion of persons with a positive family history. With 221 persons included in the ZInEP “early recognition-sample” we aim to improve individual risk assessment by developing an optimized prediction model. Focusing in addition on the putative “at-risk bipolar” group, we will have the opportunity to test the proposed risk criteria for bipolar disorder.

The development of at-risk criteria for first-episode psychosis is advanced in comparison to the developmental stage of the at-risk criteria for first-episode mania. Currently, there is no consensus on the definition of the bipolar prodrome (111). Further investigation of subthreshold symptoms is needed to identify potential prodromal symptoms. After completing the follow-up period it will be possible to refine the risk criteria for true “at-risk bipolar” individuals. Adding, e.g., imaging or neuropsychological data could further increase their predictive power (112). The bipolar-spectrum concept may also provide research for affective disorders by facilitating identification of early stage bipolar disorder (113). The concept, as suggested by Angst et al., comprises a continuum of severity and a continuum from depression to mania, providing a more differentiated research model for affective disorders. This corresponds to the genetic findings as quoted previously.

A dimensional approach with a focus on the major components of these disorders, i.e., mania, psychosis (and depression) could be a helpful strategy to disentangle subjects at-risk for mania and those at-risk for psychosis. Examinations of the familial aggregation patterns of the core components of psychosis, mania, and major depression suggest a strong familial specificity (114). Familial aggregation of bipolar disorder seems to be attributed to mania (115). Family studies demonstrated the independence of the familial transmission of mania and depression (114, 115).

Age also seems to play an important role in the distribution pattern of the different risk states with assumed different trajectories. In our sample, we will be able to examine, if the “adolescent risk-sample” differs significantly from the “adult risk-sample” in the symptom distribution. The adolescent group may develop a higher symptom load at an earlier stage, which could correlate with marked increases in neurobiological changes. Besides the on-going follow-up examination, we will analyze our recent data regarding potential correlations with data from genetic, neuropsychology, electrophysiology, MRI, sociophysiology/social cognition, stigma, resilience, stress coping, and environmental factors.

Up to now in our sample risk group, affiliation and transition to psychosis were predicted by different neuropsychological deficits, which also had a profound effect on an individual’s level of general functioning and satisfaction with life (116).

Addington and Heinssen point out that an improved prediction model should also include biomarkers (117). Using refined prediction models will help understand the development of psychotic disorders. But taking into account that a subgroup, even if they do not develop full-blown psychosis, does not necessarily experience complete remission of symptoms or improvement in functioning either, it is necessary to consider the possibility of being at-risk for mental disorders other than psychosis (118).

Also, improved prediction models encourage research on early intervention in persons with a higher risk for psychosis. Understanding the biological and environmental mechanisms involved in the onset of psychosis will help to discover potentially disease-modifying interventions. Trying to overcome problems, which arise due to categorical diagnosis, augmentation with symptom, time, severity, and persistence dimensions were recently suggested (119). Their proponents state that a prevention-oriented framework for evaluation of interventions can be provided by defining discrete stages according to progression of disease.

But early detection could unintentionally entail the “mental illness” label and may negatively affect service use. In our sample stronger self-labeling and less stigma stress predicted better attitudes toward psychiatric medication and psychotherapy. In addition, stigma stress, but not the level of perceived public stigma, predicted more negative attitudes toward help-seeking (120). Perceived public stigma appears to be associated with reduced well-being among young people at-risk of psychosis (121). Well-being is influenced moreover by changes of self-labeling and stigma stress over time independent of baseline levels (122). A non-stigmatizing use of (self-) labeling could reduce stigma stress and its impact on young people at-risk for psychosis (120). This highlights the need for specific interventions regarding stigma.

Despite the considerable evidence, which has been accumulated on early recognition of psychosis, there is a shortage of research findings regarding (a) the underlying biological mechanisms, (b) valid risk criteria for bipolar disorder, and (c) the developmental trajectories. The ZInEP “early recognition”-study enables to test the possible contributions of this multi-level-approach and thereby to contribute to an improved prediction.

## Author Contributions

The international advisory board includes Gerd Glaeske, Thomas Becker, Andreas Fallgatter, Felix Gutzwiller, Joachim Klosterkötter, Christoph Lauber, Roselind Lieb, Ulrich Meise, Hans Joachim Salize, and Wolfgang Wölwer. Anastasia Theodoridou, Karsten Heekeren, Helene Haker, Wolfram Kawohl, Nicolas Rüsch, Susanne Walitza, and Wulf Rössler designed the study. Diane Dvorsky, Sibylle Metzler, and Maurizia Franscini acquired the data. Anastasia Theodoridou drafted and revised the manuscript, which all authors reviewed and approved for publication.

## Conflict of Interest Statement

The authors declare that the research was conducted in the absence of any commercial or financial relationships that could be construed as a potential conflict of interest.
